# Effect of Tetrodotoxin Pellets in a Rat Model of Postherpetic Neuralgia

**DOI:** 10.3390/md16060195

**Published:** 2018-06-05

**Authors:** Bihong Hong, Jipeng Sun, Hongzhi Zheng, Qingqing Le, Changsen Wang, Kaikai Bai, Jianlin He, Huanghuang He, Yanming Dong

**Affiliations:** 1 Department of Materials Science and Engineering, College of Materials, Xiamen University, Xiamen 361005, China; 2 Engineering Research Center of Marine Biological Resource Comprehensive Utilization, Third Institute of Oceanography, State Oceanic Administration, Xiamen 361005, China; jpsun@tio.org.cn (J.S.); qingqingle@163.com (Q.L.); w1277027075@163.com (C.W.); kkbai@tio.org.cn (K.B.); jlhe@tio.org.cn (J.H.); 18259221714@163.com (H.H.); 3 School of pharmaceutical Sciences, Xiamen University, Xiamen 361102, China; 32320171153272@stu.xmu.edu.cn; 4 College of Chemical Engineering, Huaqiao University, Xiamen 361021, China

**Keywords:** postherpetic neuralgia, tetrodotoxin, TTX pellets, pharmacokinetics, varicella-zoster virus

## Abstract

Postherpetic neuralgia (PHN) is nerve pain caused by a reactivation of the varicella zoster virus. Medications are used to reduce PHN but their use is limited by serious side effects. Tetrodotoxin (TTX) is a latent neurotoxin that can block neuropathic pain, but its therapeutic index is only 3–5 times with intravenous or intramuscular injection. Therefore, we prepared oral TTX pellets and examined their effect in a rat model of PHN induced by resiniferatoxin (RTX). Oral TTX pellets were significantly effective at preventing RTX-induced mechanical and thermal allodynia, and similar to pregabalin. Moreover, oral administration of TTX pellets dose-dependently inhibited RTX-induced PHN compared with intramuscular administration of TTX injection. We also studied the pharmacokinetic profile of TTX pellets. Our results showed that the blood concentration of TTX reached a maximum plasma concentration (C_max_) at around 2 h, with an elimination half-life time (t_1/2_) of 3.23 ± 1.74 h after intragastric administration. The median lethal dose (LD_50_) of TTX pellets was 517.43 μg/kg via oral administration to rats, while the median effective dose (ED50) was approximately 5.85 μg/kg, and the therapeutic index was 88.45. Altogether, this has indicated that oral TTX pellets greatly enhance safety when compared with TTX injection.

## 1. Introduction

Varicella-zoster virus (VZV) is a neurotropic herpes virus. Infection by VZV usually triggers varicella, although VZV can become latent in cranial nerves and the dorsal root along the entire neuraxis, including autonomic ganglia [1]. Based on VZV gene expression [2], two severe complications of VZV infection have been shown: vasculopathy and postherpetic neuralgia (PHN). PHN is defined as severe pain occurring 1 month after rash onset or which persists for greater than 3 months [3]. Incidence of PHN among patients who have had herpes zoster may be as high as 10–35% [4]. PHN causes impaired activity including sleep disturbances, depression, and social withdrawal, which patients may suffer for years and consequently has a major influence on quality of life [5]. Currently, treatments for PHN include pharmacological therapy and minimally invasive treatments (e.g., nerve block and surgery). Traditional therapeutic drugs include anticonvulsants, opioids, tricyclic antidepressants, and non-steroidal anti-inflammatory drugs, which can relieve neuropathic pain [6]. Capsaicin or topical anesthetics (e.g., lidocaine gel) are used for mild pain. Gabapentin and pregabalin are oral anticonvulsant medications, while tricyclic antidepressants reduce PHN pain. These drugs may control the affected person’s symptoms but there is no well-established PHN modifying treatment. Further, the efficacy of these traditional drugs is often limited both in amplitude and duration, and their use is frequently associated with side effects that significantly impair quality of life for patients [7]. Tetrodotoxin (TTX) is a latent neurotoxin that can block voltage-gated sodium channels in nerves with a high degree of selectivity [8]. Indeed, a few studies have reported that TTX can suppress action potentials in axons and reduce ectopic peripheral nerve activity, which may block neuropathic pain with minimal side effects at low doses [9]. Importantly, although TTX dosages of 0.1 and 0.3 μg produced a small, albeit significant, decrease in mechanical nociceptive threshold, TTX at 1 μg did not show a significant change. Furthermore, no gross motor or behavioral side effects were reported after intramuscular (i.m.) administration of 1 μg TTX [10].

Pellets are the most commonly used drug form for their distinct advantages, such as controlled drug release, reduced local concentration, increased gastrointestinal transit time, and lower risk of dose dumping compared with tablets. TTX can improve the local anesthesia time of bupivacaine dexamethasone pellets with no in vitro cell toxicity or cell death [11]. Specifically, 0.05% *w*/*w* of TTX extended local anesthesia time from 31.3 h to 221.7 h when added to bupivacaine dexamethasone pellets. However, the potential application of TTX may be limited by toxicity, as 0.1% *w*/*w* TTX causes death in all animals [12].

At present, there are three models for evaluating the curative effects of PHN: VZV [13], herpes simplex virus [14], and resiniferatoxin (RTX) [15]. Based on the strong feasibility of RTX and its possible mimic of the PHN-like symptoms, we decided to use RTX to simulate PHN.

Here, based on analgesia of TTX and the advantage of pellets, oral TTX pellets were prepared. Next, we compared the effect of TTX pellets and TTX injection using a rat model of PHN induced by RTX. We also examined the median lethal dose (LD_50_) and pharmacokinetics of the TTX pellets. Our findings indicate that oral TTX pellets greatly enhance safety compared with TTX injection.

## 2. Results

### 2.1. Uniformity of TTX Pellets Content

The preparation of TTX pellets is described in Materials and Methods. The content of TTX in the pellets was enough to be determined by the HPLC-FLD method, as described in Materials and Methods. The HPLC-FLD method showed a good linearity in a TTX concentration range of 0.1024–10.24 μg/mL, with 0.1024 μg/mL of the lower limit of quantification (LLOQ) and 0.05 μg/mL of the limit of detection (LOD). The recovery of TTX was 98.07% ± 0.94%, 100.99% ± 0.25%, and 99.95% ± 0.55% (*n* = 3) at the 4.04, 4.92, and 3.00 μg/mL of concentration, respectively. The results showing uniformity of TTX pellets content are shown in Table 1. Average content >99% and relative standard deviation (RSD) of 1.83%, indicate homogeneous pellets content.

### 2.2. Percentage of TTX Pellets Released In Vitro

**Figure 1:**
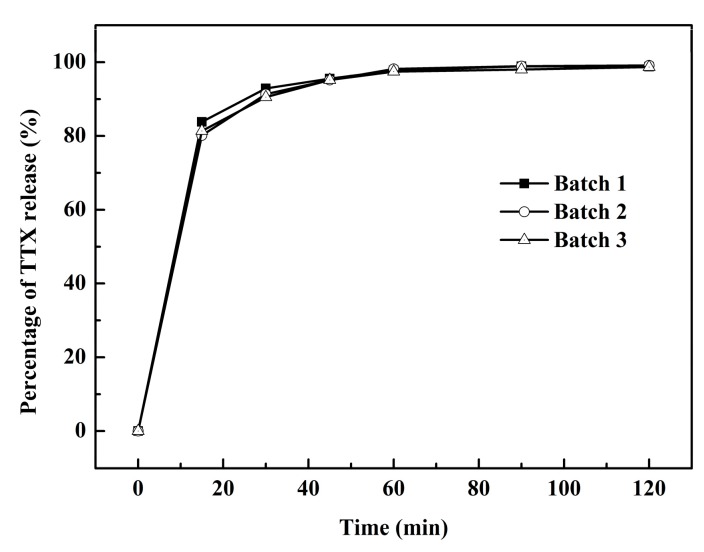
Percentage of tetrodotoxin (TTX) release in vitro.

Preparation of the three batches of TTX pellets were as described in the Materials and Methods. The amount of TTX released from the TTX pellets was enough to be determined by the HPLC-FLD method. The HPLC-FLD method showed a good linearity in a TTX concentration range of 0.5005–12.024 μg/mL, with 0.5005 μg/mL of LLOQ and 0.26 μg/mL of LOD. The recovery of TTX was 100.03% ± 0.46%, 99.67% ± 0.70%, and 100.50% ± 0.37% (*n* = 3) at the 4.00, 4.95, and 5.95 μg/mL, respectively. As shown in Figure 1, the percentage of main drug release was approximately 90% over 30 min. With increasing time, the percentage of TTX release improved up to 98% in 120 min. After this, there were no significant difference with any of the three batches.

### 2.3. Effect of TTX Pellets in a RTX-Induced Rat Model

#### 2.3.1. Effect of RTX on Mechanical and Thermal Allodynia

**Figure 2:**
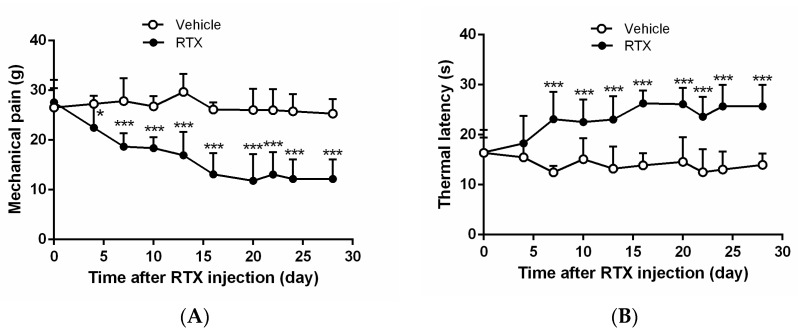
Time course of the effect of resiniferatoxin on mechanical and thermal allodynia. Time course of the mechanical withdrawal threshold in response to the von Frey filaments in vehicle- and resiniferatoxin (RTX)-treated rats (**A**); Time course of paw withdrawal latency to a noxious heat stimulus in vehicle- and RTX-injected rats (**B**). Data are expressed as mean ± SD (*n* = 10 rats in each group). * *p* < 0.05 and *** *p* < 0.001 compared with the vehicle group.

The effect of administration of RTX (200 µg/kg) on mechanical pain (Figure 2A) and thermal allodynia (Figure 2B) was examined. Prior to treatment, the mechanical withdrawal threshold was measured using von Frey filaments [16] was 27 g (*n* = 20). However, within 3 days, the mechanical threshold decreased significantly (*p* < 0.05) in all RTX-injected rats compared with vehicle-treated rats (Figure 2A). Meanwhile, paw withdrawal latency to heat stimulus increased significantly within 6 days (*p* < 0.001) in RTX-injected rats (Figure 2B). Both changes lasted for the duration of the experiment in all rats.

#### 2.3.2. Effect of TTX Pellets on Mechanical and Thermal Allodynia Induced by RTX

**Figure 3:**
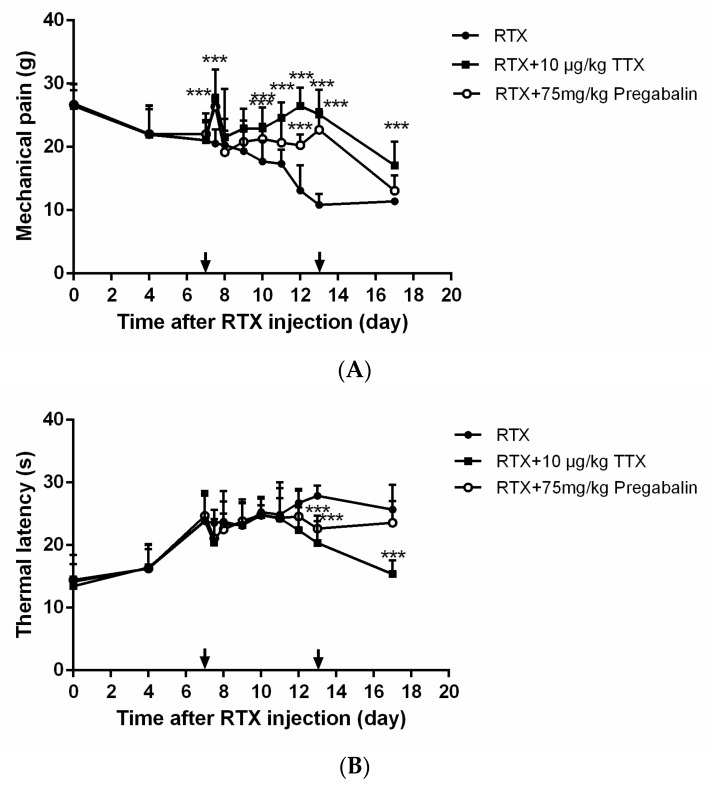
Time course of the effect of tetrodotoxin on resiniferatoxin-induced mechanical (**A**) and thermal (**B**) allodynia. Tetrodotoxin (10 μg/kg), in parallel, pregabalin (75 mg/kg) were intragastrically administered once a daily for 7 days starting from the 7th day after resiniferatoxin injection, as indicated. Withdrawal was observed from the 7th to 17th day. Mechanical withdrawal and thermal pain thresholds were detected at 1 h after TTX or pregabalin administration. Data are expressed as mean ± SD (*n* = 10). *** *p* < 0.001 compared with the RTX group.

The mechanical withdrawal threshold results are shown in Figure 3A. Seven days after the RTX injection, the mechanical withdrawal threshold increased markedly upon first administration of TTX and pregabalin. Subsequently, the mechanical withdrawal threshold decreased after TTX and pregabalin injection until the 12th or 13th day. After this, efficacy improved and was lower than the original mechanical pain.

Conversely, there was an obvious reduction in thermal latency by the 7th day after TTX or pregabalin administration (Figure 3B). The effect of both TTX and pregabalin on thermal-increased remission was not observed before 11 days. After that, TTX showed higher efficacy on thermal latency than pregabalin, which may be due to the insensitivity of RTX to thermal latency.

#### 2.3.3. Effect of TTX Pellets at Different Doses on Mechanical Allodynia

**Figure 4:**
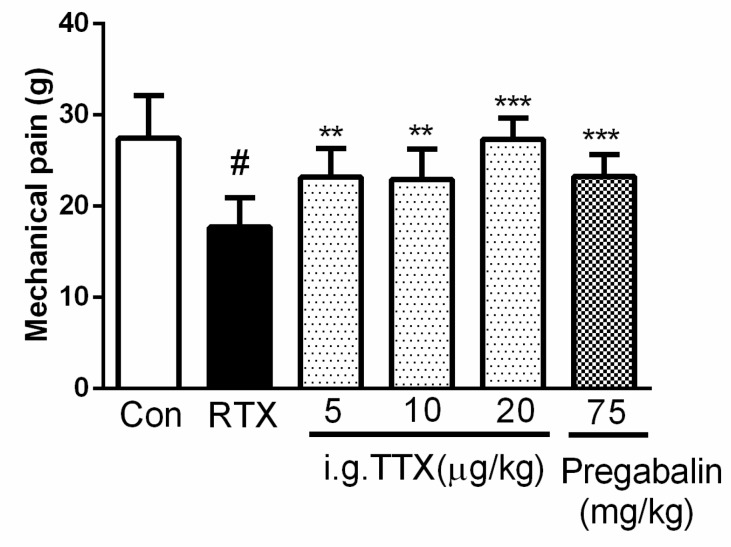
Dose response of oral tetrodotoxin on resiniferatoxin-induced mechanical allodynia. TTX was administered orally daily at doses of 5, 10, and 20 μg/kg for 4 days, starting from the 7th day after resiniferatoxin (RTX) injection. Pregabalin (75 mg/kg) was administered within the same time frame as TTX. Mechanical pain was detected at 1 h after TTX or pregabalin. Data are expressed as mean ± SD (*n* = 10). ** *p* < 0.01, *** *p* < 0.001 compared with the RTX group; ^#^ *p* < 0.05 compared with the control group.

To determine the effect of the TTX dosage on mechanical pain, rats were treated orally with various doses of TTX or pregabalin (75 mg/kg, an appropriated dosage chosen by the preliminary test with 300, 150 and 75 mg/kg.) (Figure 4). Both groups were intragastrically (i.g.) administered the drugs daily for 4 days, starting from the 7th day after RTX injection. Compared with the model group, TTX-treated rats showed a significant increase in mechanical pain response in a dose-dependent manner. In addition, a high dose of TTX (20 μg/kg) was enough to completely abolish the pain response, and pregabalin showed a similar effect at the 75 mg/kg. The median effective dose (ED50) of the oral TTX pellets was 5.85 μg/kg, as calculated by the logit method.

#### 2.3.4. Effect of TTX Pellets and TTX Injection on Mechanical Allodynia

**Figure 5:**
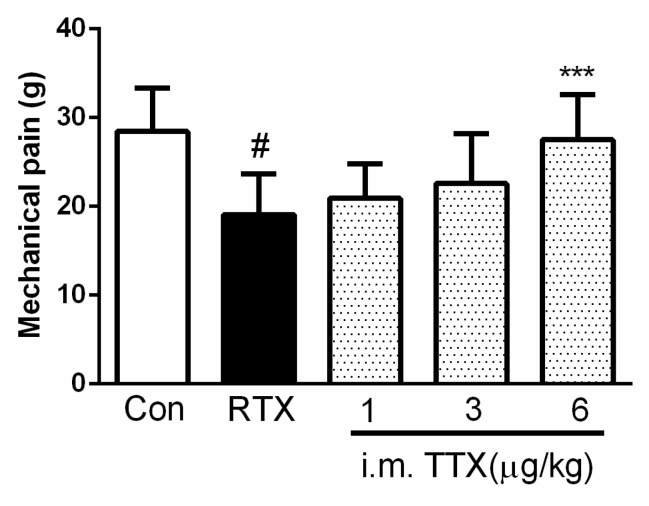
Dose response of intramuscular injection of tetrodotoxin on resiniferatoxin (RTX) -induced mechanical allodynia. TTX was injected daily for 4 days, starting from the 7th day after RTX injection. Mechanical pain was detected at 1 h after TTX injection. Data are expressed as mean ± SD (*n* = 10). *** *p* < 0.001 compared with the RTX group; ^#^ *p* < 0.05 compared with the control group.

Next, we compared the effect of the TTX pellets and TTX injection on their ability to prevent mechanical allodynia induced by RTX. As mentioned, rats were administered TTX daily for 4 days, starting from the 7th day after RTX injection. This also showed a significant increase in mechanical pain response in a dose-dependent manner. Further, TTX-oral (20 μg/kg) administration of the pellets (Figure 4) showed similar effect to that of the high dose of TTX-intramuscular injection (6 μg/kg)(Figure 5). TTX at 1 μg/kg and 3 μg/kg had no obvious effect on mechanical allodynia. ED50 of TTX injection was 2.49 μg/kg, as calculated by the logit method.

### 2.4. Acute Toxicity of TTX Pellets

Rats treated with 692 μg/kg and 814 μg/kg oral TTX pellets showed visibly less movement as well as wheezing and sluggishness. Death initially occurred at 8–10 min after administration. However, most death occurred within 2–6 h but up until 10 h, after which there was no more death. There were no obvious changes in the gross anatomy of the dead animals. As shown in Table 2, LD_50_ was 517.43 μg/kg, as calculated by the Bliss method, with a 95% confidence limit of 459.41–582.79 μg/kg. LD_50_ for female rats was 441.2 μg/kg, while for male rats was 573.95 μg/kg.

Body weight changes in Sprague–Dawley rats following a single oral administration of TTX pellets are shown in Table 3. The distribution of animal weight before the drug administration was average. No significant differences were found with increasing weight of the rats.

### 2.5. Pharmacokinetics Following Intragastric TTX Pellets Administration

#### 2.5.1. TTX Injection

The content of TTX in the blood of tested rats could only be determined by the UPLC-MS/MS method, instead of the HPLC-FLD method used for the determination of the university of TTX pellets and the release of TTX from the pellets, because of the lower level of TTX in the rat blood. The UPLC-MS/MS method showed a good linearity in a TTX concentration range of 0.02–2.00 ng/mL, with 0.02 ng/mL of LLOQ and 0.009 ng/mL of LOD. The recovery of TTX was 97.10% ± 4.52%, 92.80% ± 3.53%, and 103.00% ± 6.89% (*n* = 3) at the 0.04, 0.40, and 1.60 ng/mL, respectively.

**Figure 6:**
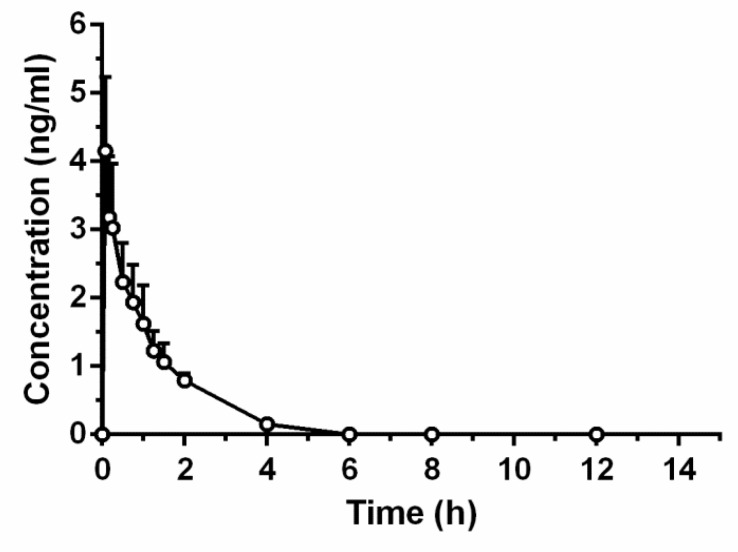
Mean concentration–time profile of tetrodotoxin in rat plasma following intravenous administration at a dose of 6 μg/kg.

The time course of TTX concentration in the blood of rats after intravenous (i.v.) injection at a dose of 6 μg/kg is shown in Figure 6. The blood concentration of TTX was 4.12 ng/mL at 0.08 h after administration and then declined gradually.

The pharmacokinetic parameters are summarized in Table 4. After i.v. injection, the mean value for systemic clearance (CL) was 1349.40 ± 326.75 mL/h/kg, with an elimination half-life time (t_1/2_) of 0.92 ± 0.17 h. The area under the plasma concentration–time curve from zero to the end time point (AUC_0–t_) and area under the plasma concentration–time curve from zero to infinity (AUC_0–∞_) was 4.42 ± 0.90 ng·h/mL and 4.63 ± 0.90 ng·h/mL, respectively, while the apparent volume of distribution (Vd) was 1824.68 ± 709.84 mL/kg.

#### 2.5.2. Intragastric TTX Pellets

**Figure 7:**
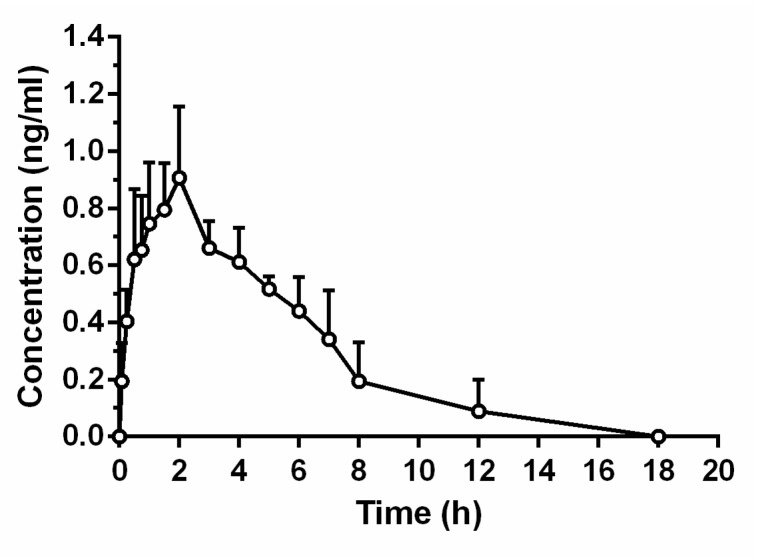
Mean concentration–time profile of tetrodotoxin pellets in rat plasma following intragastric administration at a dose of 100 μg/kg.

The pharmacokinetic profile following oral administration of 100 μg/kg (TTX could be better detected in blood) TTX pellets to rats is shown in Figure 7, with the pharmacokinetic parameters listed in Table 5. The blood concentration of TTX increased from 0 ng/mL to 0.70 ng/mL within 15 min after administration. Moreover, plasma concentration increased from 15 min to 120 min but the slope declined, after which there was a gradual increase to the peak concentration of 0.93 ± 0.23 ng/mL at 2.08 ± 0.49 h. After this, TTX concentration gradually decreased but was still present at a reasonable concentration compared with i.v. injection.

Following i.g. TTX administration, AUC_0–t_ was 4.91 ± 0.99 ng·h/mL while AUC_0–∞_ was 5.82 ± 1.65 ng·h/mL. Meanwhile, t_1/2_ was 3.23 ± 1.74 h with CL of 18188.62 ± 4234.34 mL/h/kg, showing a long-lasting effect of i.g. administration compared with i.v. injection. Further, Vd was 76276.28 ± 22601.44 mL/kg, indicating centralized organizational distribution following i.g. administration. Compared with i.v. administration of TTX, oral bioavailability of TTX pellets was only 6.7%.

## 3. Discussion

A strongly acidic condition affects the heat stability of TTX. However, TTX is relatively stable in gastric pH (1–2) around 37 °C. Following oral administration of TTX, LD_50_ was 38.12-times greater than after i.v. injection, which reflects a considerable decrease in bioavailability following oral administration [17]. Consequently, clinical injection is commonly administered through local muscle. However, although i.m. injection of TTX has a significant effect, the TTX dose is regarded as a crucial safety factor because of the high toxicity of TTX. Acute toxicity symptoms of TTX include central nervous system depression and neuromuscular blockage both locally and systemically. Acute toxicity tests in male and female rats following a single i.m. injection showed LD_50_ values of 10.53 and 11.75 μg/kg, respectively. In addition, the half-life of a muscle injection is short, with TTX only present in the plasma for 2 h. Thus, to maintain drug efficacy, frequent injections are needed, which leads to poor clinical compliance. Accordingly, an oral preparation with an extended therapeutic window (as well as clinical compliance) is necessary for the TTX application.

Subcutaneous injection of TTX has been researched for cancer-related pain. Patients who received 30 μg TTX twice daily for 4 consecutive days resulted in an analgesic response of 56.7 days, while the placebo only enabled 9.9 days [18]. There was still a significant anti-hyperalgesia effect at 24 h with topical injection of 0.03–1.0 μg /kg TTX [10]. TTX as an effective treatment at low doses has shown that 1.0–3.0 μg/kg TTX reduces the expression of mechanical allodynia. In contrast, 3.0–6.0 μg/kg TTX had no effect on the same thermal and mechanical stimuli [19].

In this study, we compared different routes of administration. Oral administration showed a flatter change, while efficacy changed rapidly with the increasing concentration of the i.m. injection. This indicates that the oral administration of TTX has a wider and safer dosage range. In addition, LD_50_ and ED_50_ of oral administration of TTX pellets were 517.43 μg/kg and 5.85 μg/kg, respectively, resulting in a therapeutic index of 88.45. In contrast, LD_50_ and ED_50_ for i.m. administration were approximately 11 μg/kg and 2.49 μg/kg, respectively, with a therapeutic index of 4.42. This further indicates the superior safety of oral administration. Nevertheless, oral administration showed a low bioavailability of approximately 6.7%, with high Vd of 76,276 mL/kg, reflecting a concentrated distribution. Some studies have reported the higher distribution of TTX in the gastric intestinal wall, lungs, kidney, and heart than in plasma at 0.5 h after i.m. administration. Meanwhile, TTX has also been shown to be rapidly distributed in the gastrointestinal tract after entering the body [20], suggesting that sodium channels in the gastrointestinal tract are blocked, which likely prolonged the duration of TTX absorption. This may be related to the long-term efficacy of oral administration. In addition, the LD_50_ for the oral single administration of TTX pellets in the female and male rats was 441.2 and 573.95 μg/kg, respectively, while the LD_50_ for the single intramuscular administration of TTX injections was 10.53 and 11.75 μg/kg in the male and female rats, respectively. This has suggested that the toxicity of TTX was different in females and males, and the females are likely more sensitive to the toxic effect of TTX than the males. The specific reasons for the differences require further detailed investigation in future studies.

To the best of our knowledge, up to date pregabalin and gabapentin were the most valuable medications in clinical use for PHN, however, there were daily episodes of somnolence that typically occurred in 25% and dizziness that occurred in 46% at the clinic [21,22]. TTX as an analgesics was applied for various pain in clinical study [23]. TTX, as an analgesic agent against uncontrolled cancer pain has been investigated in a phase III trial by WEX Pharmaceuticals. Furthermore, TTX was also evaluated to treat the neuropathic pain caused by chemotherapy-induced peripheral neuropathy in a phase II trial [24] . The analgesic effect of TTX increased in the 4 or 5th day of administration but decreased in the 10th day. Furthermore, the analgesic effect disappeared from the next week of administration. During the TTX administration, the neurological examination, vital signs, oxygen saturation, clinical chemistry tests, and 12-lead ECG measures were not affected [25]. In these clinical studies, when TTX (30 μg) was subcutaneously injected twice daily for continuous 4 days, most side effects were those with mild to moderately severities, such as tingling, numbness or other transient sensory symptoms, but no evidence of cumulative toxicity or tolerability in the long-term. In the present study, the highest dosage of TTX (20 μg/kg) of the pellets was orally administrated to rats once a day for continuous 7 days and no obvious side effects were observed in the test rats. The dosage of TTX (20 μg/kg) in the animal test was equivalent to the dosage of TTX (55.5–222.2 μg/person) in human use. Thus, the result suggested the lower side effects of the oral administration of TTX pellets than the intramuscular or subcutaneous administration of TTX injections.

## 4. Materials and Methods

### 4.1. Reagents

Tetrodotoxin standard (assay 99.2%) was provided by the Third Institute of Oceanography State Oceanic (Xiamen, China). Tetrodotoxin (assay 98.5%) and Tetrodotoxin injection (assay 10.1 μg/mL) were provided by Xiamen Chaoyang Biological Engineering Co., Ltd. (Xiamen, China). RTX was purchased from J&K Chemical (Shanghai, China). Sugar sphere (600–710 μm) was purchased from JRS PHARMA Co., Ltd. (Rosenberg, Germany). Opadry (YS-1-7006) was purchased from Colorcon Co., Ltd. (Shanghai, China). Hydroxypropyl Methylcellulose (HPMC) was purchased from Huzhou Zhanwang Pharmaceutical Co., Ltd. (Huzhou, China). Citric acid was purchased from TaishanXinning Pharmaceutical Co., Ltd. (Taishan, China). 1-Octanesulfonate sodium was provided by Regis Technologies (Morton Grove, IL, USA). Sodium phosphate monobasic dehydrate, sodium phosphate dibasic dehydrate, sodium hydroxide, phosphoric acid, and acetonitrile were provided by Sinopharm Chemical Reagent Co., Ltd. (Shanghai, China). Talc (10 μm) was purchased from Guangxi Longsheng Huamei Talc Development Co., Ltd. (Sanmen Town, China).

### 4.2. Animals and Experimental Design

#### 4.2.1. Animals

Experiments were performed using adult Sprague–Dawley rats (220–280 g) purchased from the Experimental Animal Center of Shenyang Pharmaceutical University (Shenyang, China). All procedures were approved by the Animal Care Committee at Shenyang Pharmaceutical University. Rats were acclimatized to the laboratory for at least 3 days before starting experiments. Rats were housed with five per cage on a 12h light/dark cycle, with free access to food and water under controlled temperature (20–24 °C) and humidity (45–65%). Food and water were allowed ad libitum during the study period.

#### 4.2.2. Experimental Design for RTX-Induced PHN

Each rat in the RTX group received a single intraperitoneal injection of RTX (200 μg/kg) under halothane anesthesia (2% in oxygen). RTX was dissolved in a mixture of 10% Tween 80 and 10% ethanol in normal saline. Separate rats received a mixture of 10% Tween 80 and 10% ethanol in normal saline and were used as the vehicle control. Before RTX or vehicle treatment, the baseline sensitivity of each rat to mechanical and thermal stimulation was measured.

#### 4.2.3. Nociceptive Behavioral Tests

Behavioral tests were performed three times before RTX injection, and once every other day, starting from 1 week after the RTX injection. Animals were habituated to the testing environment for 30 min. Thermal sensitivity was assessed by exposing the mid-plantar surface of the hind paw to a radiant heat beam through a transparent glass surface using a plantar analgesia meter (RWD Life Science Inc., Shenzhen, China). Mean value for withdrawal latency was calculated on two to three consecutive trials. A cutoff of 30 s was used to avoid potential tissue damage.

Mechanical allodynia was assessed by placing the rats on an elevated mesh floor, with the tactile threshold measured using the “up-down” method [26]. After an acclimation period of 30 min, a series of calibrated von Frey filaments were perpendicularly applied to the plantar surface of both hind paws with sufficient force to bend the filament for 6 s. Brisk withdrawal or paw flinching was considered a positive response. The test was repeated two to three times in each rat, and the mean value calculated.

#### 4.2.4. Statistical Analysis

All behavioral data are presented as mean ± SD. Paw withdrawal threshold or latency and the effect of TTX on the withdrawal threshold were examined by an analysis of variance followed by Tukey’s post hoc test. *p* < 0.05 was considered statistically significant.

### 4.3. TTX Pellets Content

High-performance liquid chromatography analytical method (HPLC-FLD) [27,28,29,30,31,32].

High-performance liquid chromatography (HPLC) separation was performed on a Waters 2695 HPLC system (Waters, Milford, MA, USA) using a ZORBAX SB-C8 column (4.6 mm × 250 mm, 5 μm). The mobile phase comprised 1-octanesulfonate sodium-phosphate buffer (8.95 g sodium phosphate dibasic dehydrate, 3.90 g sodium phosphate monobasic dehydrate, and 0.27 g 1-octanesulfonate sodium dissolved in distilled water up to 500 mL with stirring and filtrating), with a flow rate of 0.3 mL/min. The column temperature was maintained at 28 °C and injected volume was 10 μL. HPLC was combined with a fluorescence detector (2475; Waters), with intensity monitored using excitation and emission wavelengths of 365 nm and 510 nm, respectively. The post column reaction (reaction coil: Waters RXN 1000, Volume 1000 μL, Coil 0.018 inch ID polytetrafluoroethylene tubing knitted) reagent was 4 mol/LNaOH, injection volume of 10 μL at a flow rate of at 0.3 mL/min, and temperature of 110 °C.

TTX pellets were accurately weighed and then powdered. The powder was transferred to a 50 mL volumetric flask. After ultrasonication for 20 min, diluent solution was then cooled, stirred, and filtrated. Filtrate was detected by HPLC. The sample content was calculated using the external standard method [33].

### 4.4. Release of TTX Pellets

The release determination test was performed using the Pharmacopoeia of China (CHP) and U.S. Pharmacopeia (USP) paddle apparatus [34] (RC806D; TiandaTianfa Technology, Tianjin, China), with dissolution medium (water) equilibrated at a rotational speed of 100 r/min and a temperature of 37 ± 0.5 °C. Samples of 2 mL were automatically collected at each time point and 2 mL fresh dissolution medium replenished. Samples were passed through 0.45 μm microporous membrane and filtrates assayed by HPLC to calculate drug content in the medium and percentage of the final release amount [35].

### 4.5. Preparation of TTX Pellets

#### 4.5.1. Preparation of Drug-Loaded Pellets

TTX solution of 0.4 mg/mLwas prepared with 0.1% citric acid as the assistant-solvent and 0.5% HPMC as the binder. Drug-loaded pellets were prepared using a bottom spray fluid bed(WBF-5G; Chongqing Enger Granulating & Coating Technological, Chongqing, China) [36]. Drug-loaded pellets (400 g) were used with the following experimental conditions: inlet air temperature of 40 °C, air flow rate at 150 m^3^/h, solution temperature at 25–35 °C, atomization pressure at 0.16 MPa, and rotating pump speed of 2 r/min. After drug loading was complete, the pellets were dried in the fluid bed for 15 min at 40 °C.

#### 4.5.2. Preparation of TTX Pellets by Coating

Drug-loaded pellets (380 g) were transferred into a fluid bed. The coating solution was prepared with a mixture of 5% (*w*/*w*) 190 mL opadry and 4.8 g talc using an emulsifying homogenizer (EA 200; Shanghai OuHor Mechanical Equipment, Shanghai, China) for 10 min. Next, the coating solution was passed through a 250 μm sieve and continuously stirred [37]. The experimental condition parameters for the coating process were the same as for the drug-loaded process. After coating was completed, the pellets were dried in a fluid bed for 15 min at 40 °C.

### 4.6. Pharmacokinetic Studies

#### 4.6.1. TTX Administration and Plasma Sample Collection

For pharmacokinetic studies of the TTX injection, a group of six rats (three males and three females) with jugular vein catheterization were each i.v. administered a TTX injection (6 μg/kg). Serial blood samples (0.3 mL) were collected in heparinized tubes via the jugular vein before and at time points of 0.08, 0.17, 0.25, 0.5, 0.75, 1.0, 1.25, 1.5, 2.0, 4.0, 6.0, 8.0, and 12.0 h after administration. Plasma was separated and stored frozen at −20 °C until analysis.

For pharmacokinetic studies of TTX pellets, a group of six rats (three males and three females) with jugular vein catheterization were each i.g. administered a TTX pellets (100 μg/kg). Serial blood samples (0.3 mL) were collected in heparinized tubes via the jugular vein before and at time points of 0.08, 0.25, 0.5, 0.75, 1.0, 1.5, 2.0, 3.0, 4.0, 5.0, 6.0, 7.0, 8.0, 12.0, and 18.0 h after administration. Plasma was separated and stored frozen at −20 °C until analysis.

#### 4.6.2. Chromatographic and Mass Spectrometric Conditions (UPLC/MS/MS)

##### Liquid Chromatography Conditions

Analyte separations were performed on an Acquity UPLC-1290 system (Agilent Corp., Milford, MA, USA) using a ZIC HILIC column (2.1 × 150 mm, 5 µm) equilibrated with 80% solvent A (0.04% ammonium acetate acetonitrile solution) and 20% solvent B (0.04% ammonium acetate aqueous solution) at a flow rate of 0.4 mL/min. After 6 min, a linear gradient was run to 40% solvent over 0.1 min, and maintained for 2 min, followed by a linear gradient to 80% solvent A.

##### Mass Spectrometry Conditions

An analysis of TTX in plasma samples was performed using an AB Q-trap liquid chromatography–tandem mass spectrometry (LC-MS/MS) system (ABSCIEX, Framingham, MA, USA) equipped with electrospray ionization (ESI). Quantitative analysis of TTX was performed in the positive ion mode under the following conditions: ion spray voltage at 3500 V, 350 °C, skimmer offset at 11 V, tube lens offset at 140 V, sheath gas pressure at 90 V, auxiliary gas pressure at 10 V, sweep gas flow at 40 V, collision energy at 47 eV, and scan type at MRu. AB Analyst 1.6.1 software (ABSCIEX) was used for system control and data acquisition.

#### 4.6.3. Statistical Analysis

A pharmacokinetic analysis was performed using drug and statistics software (version 2.0, Mathematical Pharmacology Professional Committee of China, Shanghai, China) to examine key parameters including maximum plasma concentration (C_max_), time to maximum concentration (T_max_), t_1/2_, AUC_0–t_, and AUC_0–∞_. Oral bioavailability (F) was measured by comparing each AUC value after i.g. and i.v. administration according to the following equation:

F = (AUC_i.g._/Dose_i.g._)/(AUC_i.v._/Dose_i.v._) × 100%

## 5. Conclusions

In the present study, we examined the effect of TTX pellets in a rat model of PHN. Oral administration of TTX pellets was more effective at preventing RTX-induced mechanical and thermal allodynia than pregabalin or TTX injection. We also determined LD_50_ after oral administration of TTX pellets to rats, showing that oral TTX pellets greatly enhance safety when compared with TTX injection.

## Figures and Tables

**Table 1:** Uniformity of tetrodotoxin pellets content (batch 2).

Sample	Content (%)	Average Content (%)	RSD (%)
1	97.02	99.21	1.83
2	97.97		
3	99.42		
4	100.02		
5	97.11		
6	99.78		
7	102.20		
8	97.16		
9	101.06		
10	100.33		

**Table 2:** Acute toxicity test showing death after a single oral administration of tetrodotoxin pellets to Sprague–Dawley rats.

Dose (μg/kg)	♀	♂	Death
814	4/5	4/5	8/10
692	3/5	3/5	6/10
588	4/5	2/5	6/10
500	3/5	3/5	6/10
425	2/5	1/5	3/10
Total	16/25	13/25	29/50
LD_50_	441.2	573.95	517.43

**Table 3:** Body weight changes of Sprague–Dawley rats after single oral administration of tetrodotoxin pellets.

Dose (μg/kg)	Gender	Body Weight	
		0 Day	7 Day
814	♂	197.6 ± 5.03	247 (*n* = 1)
	♀	188.4 ± 7.47	196 (*n* = 1)
692	♂	189.2 ± 10.62	230 ± 16.97 (*n* = 2)
	♀	189.8 ± 6.30	202 ± 5.66 (*n* = 2)
588	♂	184.8 ± 4.60	237 ± 10.82 (*n* = 3)
	♀	193.0 ± 9.17	215 (*n* = 1)
500	♂	188.8 ± 8.56	226.5 ± 7.78 (*n* = 2)
	♀	190.8 ± 9.58	199 ± 6.74 (*n* = 2)
425	♂	193.6 ± 6.54	250 ± 7.21 (*n* = 4)
	♀	187.2 ± 4.60	201.5 ± 13.44 (*n* = 3)

**Table 4:** Pharmacokinetic parameters of tetrodotoxin in rat plasma samples after intravenous administration.

Parameters	Unit	Intravenous Tetrodotoxin of 6 μg/kg
**AUC_0–t_**	ng·h/mL	4.42 ± 0.90
**AUC_0–∞_**	ng·h/mL	4.63 ± 0.90
**t_1/2_**	h	0.92 ± 0.17
**CL**	mL/h/kg	1349.40 ± 326.75
**Vd**	mL/kg	1824.68 ± 709.84

**Table 5:** Pharmacokinetic parameters of tetrodotoxin pellets in rat plasma samples after oral administration.

Parameters	Unit	Intragastric Tetrodotoxin of 100 μg/kg
**C_max_**	ng/mL	0.93 ± 0.23
**T_max_**	h	2.08 ± 0.49
**AUC_0–t_**	ng·h/mL	4.91 ± 0.99
**AUC_0–∞_**	ng·h/mL	5.82 ± 1.65
**t_1/2_**	h	3.23 ± 1.74
**CL**	mL/h/kg	18,188.62 ± 4234.34
**Vd**	mL/kg	76,276.28 ± 22,601.44
**F**		6.7%
